# To the Editors of the Medical and Physical Journal

**Published:** 1805-12

**Authors:** R. Hall

**Affiliations:** Clements Inn


### *To the Editors of the Medical and Physical Journal.*

Gentlemen,

In a communication of the 21st September, on the subject of vaccine inoculation, inserted in the Medical and Physical Journal for last month, I took occasion to lament the prejudices that have been so industriously, and but too successfully, circulated in this country against that practice; for though many have availed themselves of its salutary influence in shielding the constitution from the small-pox, it is yet certain, that several, particularly of the less enlightened orders of the community, have been deterred from its adoption, through the ill founded rumours propagated by the ignorant, and those who find their interest in decrying it. Impressed with the conviction, that such calumnies are best combated by the evidence of facts, I have transmitted for insertion in your Journal, the following extract from M. Mangourit's recent travels in Hanover; from which it appears, that after a cautious and extensive trial, by some of the most enlightened medical practitioners in Gottingen, vaccination has not only been universally adopted in that Electorate, but very generally throughout Germany, and that it continues every where to be crowned with the most compleat success. I am fully aware that we already possess ample information from various parts of the Continent, respecting this important subject, yet, I trust, it may not be deemed superfluous, to give as much publicity as possible to the statement of an intelligent traveller, who reports what he himself witnessed, unfettered by any preconceived hypothesis, and whose testimony therefore can admit neither of misrepresentation nor controversy.

"The physicians at Gottingen," observes M. Mangourit, "were eager to submit the new practice to the most rigorous examination; in consequence of which they have become fully convinced of its safety and utility. Professor Arnemann has vaccinated, gratis, the majority of the children in this city, and very few of the parents offered the least objection. M. Osiander has published an account of these experiments; the results of which are uniformly decisive in favour of the practice; in fine, all the physicians in Gottingen practise vaccination, without the smallest hesitation, and with the most compleat success. From the example afforded in this seat of the

 science sciences, the practice has been diffused throughout Hanover, and whole villages have been vaccinated. The peasants, who in general are rather averse from innovations of this nature, have however readily submitted to it, not because they are able to appreciate the evidence in its favour, but because the disease is so mild as not to prevent them pursuing their ordinary occupations. Vaccination is become so universal in every corner of the electorate, that the government have found it necessary to interfere, not with a view of interrupting the practice, but of regulating it, so far as to oblige practitioners to transmit every year to the regency, a tabular statement, containing the number vaccinated, their names, age, profession, place of residence, and the state of their healths; as well as a short account of the individual subject from which the vaccine virus was taken, the period of vaccination, its progress, and whether any maladies supervened. This regulation is yet too recent for me to speak of the consequences that may result from it; but the Germans are so fully convinced of the benefit and safety of the practice in question, that they have given to it the appellation of *schuts-blattern*, the protecting pock. The adoption of vaccination by the learned and professional men in Germany, whose opinions are never formed but in consequence of the most careful and enlarged examination, affords a more decisive proof of the efficacy of this practice than the assertions, the theories, and the most brilliant declamations of the French Physicians."

*Clements Inn,* I am, &c.

*November* 17, 1805. R. HALL.

---

